# *WDR36* variants in East Indian primary open-angle glaucoma patients

**Published:** 2011-10-08

**Authors:** Suddhasil Mookherjee, Subhadip Chakraborty, Mansi Vishal, Deblina Banerjee, Abhijit Sen, Kunal Ray

**Affiliations:** 1 Molecular & Human Genetics Division, CSIR-Indian Institute of Chemical Biology, Kolkata, India; 2 Dristi Pradip, Jodhpur Park, Kolkata, India

## Abstract

**Purpose:**

Glaucoma is a heterogeneous group of optic neuropathies with a complex genetic basis. To date, only the following four genes have been identified: viz. myocilin (*MYOC*), optineurin (*OPTN*), WD repeat domain 36 (*WDR36*), and neurotrophin 4 (*NTF4*). However, there are conflicting reports regarding the involvement of *WDR36* in the pathogenesis of primary open-angle glaucoma (POAG). In the Asian population, mutations in *WDR36* appear to play a minor role in POAG pathogenesis but polymorphic variants have been found to be associated with POAG, especially in patients with high tension glaucoma (HTG). The purpose of this study is to determine the role of *WDR36* in East Indian POAG patients. To date, no other studies have yet examined this role.

**Methods:**

Ten single nucleotide polymorphisms (SNPs; rs1971050, rs1993465, rs13153937, rs10038177, rs11241095, rs10043631, rs10038058, rs10491424, rs17553936, and rs13186912) spanning almost the entire *WDR36* gene were selected and their association with eastern Indian POAG patients was evaluated. Our study pool consisted of 323 POAG patients. Of these 116 were patients who had HTG with intraocular perssure (IOP) >21mmHg and 207 were found to be non-HTG patients (presenting IOP<21mmHg). The study also included 303 participants as controls. The polymorphisms were genotyped in both the patients and the controls using the PCR-RFLP method. Moreover, the SNP that showed significant association was validated by DNA sequencing. The haplotypes were obtained using Haploview 4.1 software. The allele and haplotype frequencies were compared between the patient group and the control group using Pearson’s χ^2^ test.

**Results:**

First, we genotyped the selected SNPs in the 323 POAG patients and 119 of the participants in the control group, in which only rs10038177 (c.710+30C>T) was found to be strongly associated with the HTG cases (OR=2.186; 95% CI=1.458–3.277; p=1.4×10^−4^). To increase the significance of the study, the SNP was genotyped in an additional 184 of the participants in the control group and it was observed that the SNP retained the association (OR=1.216; 95% CI=1.064–2.306; p=0.002). However, no haplotype was found to have any sustainable association with POAG. Based on the LD pattern and location of rs10038177, exon 5 of *WDR36* was sequenced but no suspected disease-causing variant was detected.

**Conclusions:**

Our study suggests a possible association between *WDR36* SNP in a cohort of eastern Indian POAG patients who also have high intraocular pressure (IOP). This study needs to be further validated in a larger patient cohort.

## Introduction

Glaucoma is a heterogeneous group of optic neuropathies with a complex genetic basis [1]. It diminishes vision, often without any symptoms or warning. After cataract, glaucoma is the second largest blinding disorder [2]. The latest reports estimate that, in 2010, 60.5 million people had primary glaucoma. By 2020, this number is estimated to increase to 79.6 million. This could result in bilateral blindness in 8.4 million people in 2010 and 11.2 million people by 2020 [3]. Among the three principle subtypes of glaucoma [4], primary angle closure glaucoma (PACG) is reported to occur most frequently in the Asian and African population [5,6] while primary open-angle glaucoma (POAG) is the most frequently occurring subtype in the Western population [7,8]. However, Raychaudhuri et al. [9] reported that the incidence of POAG is more frequent than the incident of PACG (10:1) in the inhabitants of West Bengal (an eastern state of India); this region is the area in which this current study was conducted.

The complexity of POAG has recently been reviewed in detail [1,10]. To date, 27 loci has been reported to be linked with POAG, but only four genes have been identified: viz. Myocilin (*MYOC*) [11], Optineurin (*OPTN*) [12], WD repeat domain 36 (*WDR36*) [13], and neurotrophin 4 (*NTF4*) [14,15].

However, the articles that have been published following the discovery of *WDR36* as a candidate for POAG suggested variable levels of involvement of the gene in the pathogenesis of this disease [16-24].

Most of the studies regarding the involvement of WDR36 in POAG have been conducted on Caucasian populations [17-20,22,23] and only two studies have been reported on Asian populations; one study reported on Japanese participants [21] and the other reported on Chinese [16] participants. In the Japanese study, S664L was identified as the only disease-causing variant in one out of the 136 HTG cases. Additionally, that study reported two SNPs (I264V and c1965–30A>G) to be associated with high tension glaucoma (HTG) [21]. In the study of the Chinese population, I713V was found to be the only potential disease-causing variant, which was identified in three out of the 82 HTG patients. Furthermore, a strong association with IVS+30 C>T (rs10038177) was observed in the HTG patients [16]. From these studies, it appears that mutations in *WDR36* play a minor role in POAG pathogenesis in Asian populations but the common variants of *WDR36* are associated with POAG, especially in patients with HTG. We performed a limited screening of *WDR36* based on previous reports and domain information but, apart from the reported single nucleotide polymorphisms (SNPs) and one synonymous change (G134G), we failed to identify any mutation in this gene [25]. Therefore, we investigated the role of *WDR36* SNPs in POAG patients and, to the best of our knowledge, no study on the role of this gene has yet been conducted on East Indian POAG patients.

## Methods

### Selection of study subjects

The patients with POAG and the control subjects were recruited for the study from Dristipradip Eye Clinic, Kolkata (West Bengal, India). The individuals speak Bengali and belong to the Indo-European linguistic group. The patient cohort consisted of 323 POAG patients among which 116 were considered to have HTG and presented with intraocular pressure (IOP) >21 mmHg and 207 were considered to be non-HTG (with presenting IOP<21 mmHg). Diagnosis of POAG involved clinical, ocular, and systemic examinations. IOP was measured using Goldmann applanation tonometry (Haag-Streit, Inc. Mason, OH) followed by pachymetry (Ocuscan A, Alcon, TX). A Goldmann 3-mirror gonioscope (Ocular Instrument, Bellevue, WA) was used to assess the angles of the anterior chamber and the optic disc. The optic disc was also evaluated with a +78 D lens. Automated threshold field analysis was conducted using the Humphrey Field Analyzer II (Carl Zeiss, Dublin, CA). The retinal nerve fiber layer (RNFL) was investigated using scanning laser polarimetry (SLP) with a variable corneal compensation technique (GDx-Vcc; Carl Zeiss, Dublin, CA).

The suspicion of POAG was raised by the presence of increased intraocular pressure above 21 mmHg, significant cupping of the optic disc (>0.7), with or without peripapillary changes, and the presence of a clinically open angle (angle of the anterior chamber) on the gonioscopy. POAG was further confirmed by typical reproducible visual field changes, viz, arcuate, Bjerrum, Seidel, paracentral and annular scotoma with nasal steps, and scanning laser polarimetry for RNFL analysis (nerve fiber Indicator>30). RNFL analysis was used to identify the pre-perimetric cases. Those patients were categorized as the HTG patients. The non-HTG group of patients who had an IOP of less than 21 mmHg on presentation but had cupping of the optic disc, showed RNFL loss as diagnosed by SLP and visual field changes characteristic of POAG. In each case, the IOP was corrected for central corneal thickness (CCT). Thus, the patient pool consisted of 323 adult onset open-angle glaucoma cases. The age at diagnosis ranged from 41 to 88 years, with a mean±standard deviation of 63.5±10.4 years. Table 1 provides the distribution of age and gender for both the patient group and the control group. Individuals with any history of inflammation or ocular trauma (past or present) and ocular hypertension were excluded from this study.

**Table 1:** Distribution of age and gender in patient and control cohorts.

**Study phase**	**Patient Subgroup**	**Number of patients**	**Average age of patients (±SD)**	**Number of controls**	**Average age of controls (±SD)**	**p- value**	**Gender distribution in patient**	**Gender distribution in control**	**p-value**
Phase 1*	POAG	323	63.5±10	119	57.3±9.7	<0.0001	M-57%, F-43%	M-50.5%, F-49.5%	0.21
Phase 1*	Non-HTG	207	65±10	119	57.3±9.7	<0.0001	M-61%, F-39%	M-50.5%, F-49.5%	0.06
Phase 1*	HTG	116	60±10	119	57.3±9.7	0.0506	M-50%, F-50%	M-50.5%, F-49.5%	0.95
Phase 2#	HTG	116	60±10	303	50.5±10	<0.0001	M-50%, F-50%	M-55%, F-45%	0.31

*In phase 1 of the study, a total 10 SNPs (rs1971050, rs1993465, rs13153937, rs10038177, rs11241095, rs10043631, rs10038058, rs10491424, rs17553936, and rs13186912) were genotyped in 323 POAG patients (207 non-HTG, 116HTG) and 119 controls. ^#^In phase 2 of the study only the SNP (rs10038177) found significantly associated in the first phase was genotyped in an additional 184 controls for providing additional power to the study.

In this study, a total of 303 participants in the control group were recruited in two phases. For 119 of the 303 controls, all the 10 SNPs were genotyped in the first phase. An additional 184 controls were genotyped for rs10038177 in the second phase. The controls were selected based on criteria which included: age >40 years (mean age±SD, 50.5±10 years; Table 1); no family history of glaucoma or ocular hypertension; IOP less than 20 mmHg in both eyes in at least one of their last two checkups; CCT greater than 500 μm in both eyes; no visual field defect; normal scanning laser polarimeter parameters (i.e., a good yellowish “bow-tie” scan pattern, a deviation map within the normal limit, a good double-hump pattern in the conduction map, temporal-superior-nasal-inferior-temporal (TSNIT) parameters within the normal limit, a nerve fiber indicator (index) <30 for both eyes); cup discs that were physiologic and similar in both eyes; a cup-to-disc ratio <0.2; no defect in disc rim or margin; and no sphincter hemorrhage around the disc. Individuals with high myopia, diabetes, and hypertension were excluded from the control group. The study protocol adhered to the tenets of the Declaration of Helsinki and was approved by the Institutional Review Board.

### Collection of blood samples and genomic DNA preparation

Eight milliliters of peripheral blood was collected with EDTA from the POAG patients and the patients without POAG with their written consent. Genomic DNA was prepared from fresh whole blood using the PAX gene blood DNA isolation kit (Qiagen, Hilden, Germany) according to the manufacturer’s protocol. The DNA was dissolved in TE (10 mM Tris-HCl, 1mM EDTA, pH 8.0).

### Genotyping

Genotyping of the patients and the control samples was conducted using PCR-RFLP methods. The sequences for primers used in PCR and the details of the RFLP analysis are shown in Table 2. The PCR conditions were as follows: an initial denaturation at 95 °C for 4 min followed by 35 cycles of 30 s of denaturation at 95 °C, 30 s of annealing at 50–62 °C and 30 s of extension at 72 °C. This was followed by a final extension at 72 °C for 4 min. All the PCR products were detected on a 6% polyacrylamide gel with ethidium-bromide staining. The PCR products were subjected to restriction digestion with appropriate enzymes from NEB (New England Biolabs, Inc. Beverly, MA) for 3 h at optimum temperatures. The digested products were analyzed on a 6% polyacrylamide gel and the alleles were scored as described in Table 2. The fidelity of the allele scoring was examined by direct sequencing of 10% of the samples in which association was observed. The results suggested complete concordance between the two methods. The sequencing reaction was conducted in a DNA sequencer (ABI 3130XL; Applied Biosystems, Foster City, CA) with BigDye® terminator chemistry.

**Table 2:** PCR primers, annealing temperature and RFLP pattern for genotyping of *WDR36* SNPs.

**SNP ID and location in the gene**	**Primer sequences**	**Annealing temperature**	**Restriction enzyme**	**RFLP pattern**
rs1971050 (c.330+925 C>T)	5′-GAGGTGAAGAGCAATTGGGTTTCTC-3′	60 °C	AluI	C: 203 bp, 35 bp T: 238 bp
	5′-GCAGTGTCAGGAAAGACACTGTACC-3′			
rs1993465 (c.459+183A>G)	5′-TTCTTTACCCAGCACACTCTGGAA-3′	56 °C	HinfI	G: 147 bp, 25 bp A: 172 bp
	5′-TTTTGAAGAAGGTCTCCAAGTGATT-3′			
rs13153937 (c.460–112A>G)	5′-GCAGATGAACATGCCTGGTCCCTTA-3′	58 °C	BciVI	G: 397 bp A: 336 bp, 61 bp
	5′-ACAGGCAAAATCTCTGGCATAG-3′			
rs10038177 (c.710+30C>T)	5′-GCCTCTCATTTATTTTATTTCTCAAGG-3′	62 °C	AluI	C: 338 bp, 120 bp T: 458 bp
	5′-CCTCTGATACAGGGGACCAACTG-3′			
rs11241095 (c 772G>A, I264V)	5′-GAGGATGGGATGTTGACTGGCAGA-3′	50 °C	HpaI	G: 268 bp, 23 bp A: 293 bp
	5′-ATAACTTGACCTGACATAAGGTTAA-3′			
rs10043631 (c.1494+90C>T)	5′-TCCCTAGTGTCTGAAATATTGGATGC-3′	50 °C	DdeI	C: 328 bp, 25 bp T: 353 bp
	5′-TAAAATGTTAAACTACGTTTTCTCA-3′			
rs10038058 (c.1494+143G>A)	5′-TCTTGTTATACTCAAGGTTTCTCATCA-3′	50 °C	ApoI	G: 191 bp, 63 bp A: 102 bp, 89 bp, 63 bp
	5′-AGAATAGTCTTTTGAACAAATAGTGCTG-3′			
rs10491424 (c.1965–905C>T)	5′-CTATCTCCTCTTAAGTGGTTAATGAATT-3′	58 °C	EcoRI	C: 147 bp, 24 bp T: 171 bp
	5′-ATGTGGTGGATAAGGCAAGG-3′			
rs17553936 (c.1965–30A>G)	5′-TGGTGACTTTCTGATCAATGCTGGTG-3′	62 °C	CvIQI	G: 308 bp, 120 bp A: 428 bp
	5′-TCAAGCAGATGTTGAATCACTCAGA-3′			
rs13186912 (c.2181A>T; V727V)	5′-GGTGTTTTGGGTCTAGTGATGG-3′	56 °C	BstBI	A: 234 bp T: 210 bp, 24 bp
	5′ -CACTTGGTTCTACTGTTTCTTTCGA-3′			

The dbSNP reference IDs for each SNP and their location in the genomic region are furnished. Ten SNPs (rs1971050, rs1993465, rs13153937, rs10038177, rs11241095, rs10043631, rs10038058, rs10491424, rs17553936, and rs13186912) were genotyped in 323 POAG patients (207 non-HTG, 116HTG) and 119 controls by RFLP analysis as mentioned in the Table.

### Bioinformatics and statistical analysis

The linkage disequilibrium (LD) among the 10 SNPs was analyzed using Haploview software (Version 4.1). Estimated haplotype frequencies were obtained by standard E-M algorithm [26]. The odds ratio (95% CI) and the p-value for each haplotype were obtained by comparing the results with all other combined haplotypes. The allele frequencies of the SNPs and the haplotypes were compared between patients in the POAG group and participants in the control group, using Pearson’s chi square test.The p-values were further corrected using the Bonferroni method. The distribution of age and gender between the patient group and the control group was compared using an unpaired two-tailed *t*-test and a two-tailed Z-test to determine the proportion, respectively.

## Results

**Figure 1:**
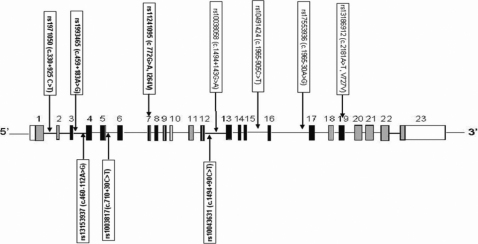
Schematic representation of *WDR36* with the location of SNPs selected for the study. The sizes of exons and introns shown in the illustration are not according to scale.

We selected 10 SNPs (rs1971050, rs1993465, rs13153937, rs10038177, rs11241095, rs10043631, rs10038058, rs10491424, rs17553936, and rs13186912) based on previous reports of the association between POAG and the SNP location, such that the SNPs spans the entire *WDR36* gene (Figure 1). In the first phase of the study, all the SNPs were genotyped in all 323 of the POAG patients and in 119 of the controls.

### rs10038177 as a risk factor for high tension glaucoma

Among the 10 SNPs, only rs10038177 (c.710+30C>T) showed a marginal association with POAG cases (age >40 years) with the C-allele portraying risk (OR=1.569; 95% CI=1.107–2.225; p=0.011; Table 3). However, when the patient pool was divided into two groups, i.e., HTG and non-HTG groups based on the presenting IOPs, the SNP showed a strong association with HTG (OR=2.186; 95% CI=1.458–3.277; p=1.4×10^−4^) with C-allele as a risk factor, which was sustained after applying a Bonferroni correction for multiple tests (Table 3).

**Table 3:** Allele frequency in POAG patients and controls.

**SNP ID and location in *WDR36***	**Allele**	**Patient subgroup**	**Frequency in patients (number)**	**Frequency in controls (number)**	**p-value**	**Bonferroni corrected p-value**	**OR (95% CI)**	**Relative risk (95% CI)**
rs1971050 (c.330+925 C>T)	C	POAG	0.10 (67)	0.09 (21)	0.495	-	-	-
		HTG	0.13 (30)		0.152	-	-	-
		NON-HTG	0.09 (37)		0.961	-	-	-
	T	POAG	0.90 (579)	0.91 (217)	0.495	-	-	-
		HTG	0.87 (202)		0.152	-	-	-
		NON-HTG	0.91 (377)		0.961	-	-	-
rs1993465 (c.459+183A>G)	A	POAG	0.35 (225)	0.32 (76)	0.420	-	-	-
		HTG	0.40 (93)		0.066	0.66	-	-
		NON-HTG	0.32 (132)		0.990	-	-	-
	G	POAG	0.65 (421)	0.68 (162)	0.420	-	-	-
		HTG	0.60 (139)		0.066	0.66	-	-
		NON-HTG	0.68 (282)		0.990	-	-	-
rs13153937 (c.460–112A>G)	A	POAG	0.29 (185)	0.24 (57)	0.166	-	-	
		HTG	0.23 (53)		0.777	-	-	
		NON-HTG	0.32 (132)		0.032	0.32	1.486 (1.035–2.133)	1.147 (1.007–1.287)
	G	POAG	0.71 (461)	0.76 (181)	0.166	-	-	-
		HTG	0.77 (179)		0.777	-	-	-
		NON-HTG	0.68 (282)		0.032	0.32	-	-
rs10038177 (c.710+30C>T)	C	POAG	0.30 (197)	0.22 (52)	0.011	0.11	1.569 (1.107–2.225)	1.119 (1.023–1.209)
		HTG	0.32 (88)		0.00014*	0.0014	2.186 (1.458–3.277)	1.440 (1.190–1.712)
		NON-HTG	0.26 (109)		0.202	-	-	-
	T	POAG	0.70 (449)	0.78 (186)	0.011	0.11	-	-
		HTG	0.68 (144)		0.00014*	0.0014	-	-
		NON-HTG	0.74 (305)		0.202	-	-	-
rs11241095 (c 772G>A, I264V)	A	POAG	0.62 (399)	0.62 (148)	0.909	-	-	-
		HTG	0.65 (151)		0.513	-	-	-
		NON-HTG	0.6 0 (248)		0.566	-	-	-
	G	POAG	0.38 (247)	0.38 (90)	0.909	-	-	
		HTG	0.35 (81)		0.513	-	-	-
		NON-HTG	0.40 (166)		0.566	-	-	-
rs10043631 (c.1494+90C>T)	C	POAG	0.28 (184)	0.27 (64)	0.640	-	-	-
		HTG	0.31 (72)		0.322	-	-	-
		NON-HTG	0.27 (112)		0.964	-	-	-
	T	POAG	0.72 (462)	0.73 (174)	0.640	-	-	-
		HTG	0.69 (160)		0.322	-	-	-
		NON-HTG	0.73 (302)		0.964	-	-	-
rs10038058 (c.1494+143G>A)	A	POAG	0.35 (225)	0.33 (79)	0.650	-	-	-
		HTG	0.38 (88)		0.283	-	-	-
		NON-HTG	0.33 (137)		0.976	-	-	-
	G	POAG	0.6 5(421)	0.67 (159)	0.650	-	-	-
		HTG	0.62 (144)		0.283	-	-	-
		NON-HTG	0.67 (277)		0.976	-	-	-
rs10491424 (c.1965–905C>T)	C	POAG	0.40 (262)	0.41 (98)	0.868	-	-	-
		HTG	0.36 (84)		0.269	-	-	-
		NON-HTG	0.43 (178)		0.651	-	-	
	T	POAG	0.60 (384)	0.59 (140)	0.868	-	-	-
		HTG	0.64 (148)		0.269	-	-	-
		NON-HTG	0.57 (236)		0.651	-	-	-
rs17553936 (c.1965–30A>G)	A	POAG	0.64 (414)	0.63 (151)	0.860	-	-	-
		HTG	0.66 (154)		0.505	-	-	-
		NON-HTG	0.63 (260)		0.937	-	-	-
	G	POAG	0.36 (232)	0.37 (87)	0.860	-	-	-
		HTG	0.34 (78)		0.505	-	-	-
		NON-HTG	0.37 (154)		0.937	-	-	-
rs13186912 (c.2181A>T) (V727V)	A	POAG	0.69 (446)	0.68 (162)	0.782	-	-	-
		HTG	0.73 (169)		0.257	-	-	-
		NON-HTG	0.67 (277)		0.761	-	-	-
	T	POAG	0.31 (200)	0.32 (76)	0.782	-	-	-
		HTG	0.27 (63)		0.257	-	-	-
		NON-HTG	0.33 (137)		0.761	-	-	-

*Significant observation. The dbSNP reference IDs for each SNP and their location in the genomic region are furnished. Ten SNPs (rs1971050, rs1993465, rs13153937, rs10038177, rs11241095, rs10043631, rs10038058, rs10491424, rs17553936, and rs13186912) were genotyped in 323 POAG patient (207 non-HTG, 116HTG) and 119 controls. Among the 10 SNPs analyzed only one SNP rs10038177 (c.710+30C>T) was found to be significantly associated with the entire POAG patient cohort and when it is subdivided to two groups: HTG (IOP >21 mmHg) and non-HTG (IOP <21 mmHg), rs10038177 (c.710+30C>T) was found to be strongly associated with HTG patient cohort even after Bonferroni correction for multiple tests. The weak association of rs13153937 (c.460–112A>G) was nullified after Bonferroni adjustments for multiple tests.

To strengthen the observation made for rs10038177, the SNP was genotyped in an additional 184 of the 303 total control group participants so as to match the number of POAG cases. This SNP was found to retain a strong association with the HTG patient group (OR=1.216; 95% CI=1.064–2.306; p=0.002) with an increased number of controls (Table 4). In the genotype analysis, both CC and a combined genotype of CC and CT were found to be associated with risk for the HTG patient group (Table 5). Interestingly, the combined genotype of CC and CT was found to have a more deleterious effect (OR=1.789; 95% CI=1.158–2.763; p=0.009) than the CC genotype (OR=2.306; 95% CI=1.180–4.508; p=0.014) alone.

**Table 4:** Allele frequency of rs10038177 with additional controls.

**Allele**	**Patient Subgroup**	**Frequency in Patient (no. of Chromosomes)**	**Frequency in Control (no. of Chromosomes)**	**p-value**	**OR (95% CI)**	**Relative risk (95% CI)**
C	POAG	0.30 (197)	0.27 (162)	0.141		
	HTG	0.32 (88)		0.002*	1.216 (1.064–2.306)	1.437 (1.139–1.799)
	NON-HTG	0.26 (109)		0.886		
T	POAG	0.70 (449)	0.73 (444)	0.141	-	-
	HTG	0.68 (144)		0.002	-	-
	NON-HTG	0.74(305)		0.886	-	-

rs10038177 (c.710+30C>T) was found to be strongly associated with HTG patient group in the first phase of the study. To strengthen the observation, rs10038177 was genotyped in additional 184 controls and the SNP was still found to be strongly associated with HTG patient group. *Significant observation; P-value, OR and Relative Risk has been assessed with respect to C allele.

**Table 5:** Genotype frequency of SNP (rs10038177) showing association with POAG.

**Genotype**	**Patient subgroup**	**Frequency in patients (n)**	**Frequency in controls (n)**	**p-value**	**OR (95% CI)**	**Relative risk (95% CI)**
TT	POAG	0.47 (152)	0.53 (161)	0.129	-	-
	HTG	0.39 (045)		0.009	0.559 (0.362–0.864)	0.655 (0.466–0.915)
	NON-HTG	0.52 (107)		0.748	-	-
CT	POAG	0.45 (145)	0.40 (121)	0.210	-	-
	HTG	0.47 (054)		0.219	-	-
	NON-HTG	0.44 (091)		0.365	-	
CC	POAG	0.08 (26)	0.07 (021)	0.596	-	-
	HTG	0.14 (017)		0.014*	2.306 (1.180–4.508)	1.722 (1.077–2.487)
	NON-HTG	0.04 (009)		0.223	-	-

*Significant observation. Comparison of the genotype frequencies of rs10038177 among 323 POAG (116 HTG cases and 207 non-HTG cases) patients and 303 controls revealed biased distribution for the CC genotype (p=0.014) portraying risk for HTG patient group.

Based on the association data and the extent of the LD between the different SNPs, the nearest exon of associated polymorphism rs10038177 (i.e., exon 5) was sequenced for any possible disease causing variant in 116 of the HTG patients. However, except for the SNP (rs10038177), no other variant was identified in the region.

### Lack of association between POAG and other SNPs in relation to *WDR36*

The SNP rs13153937 (c.460–112A>G) was found to be marginally associated with the non-HTG patient group (OR=1.486; 95% CI=1.035–2.133; p=0.032) with ‘G’ as the risk allele. However, the association did not sustain after the Bonferroni correction was conducted for multiple tests. Other SNPs did not show any association with the POAG patients, the HTG patients, or the patients in the non-HTG group.

### Linkage disequilibrium pattern between the SNPs in *WDR36*

**Figure 2:**
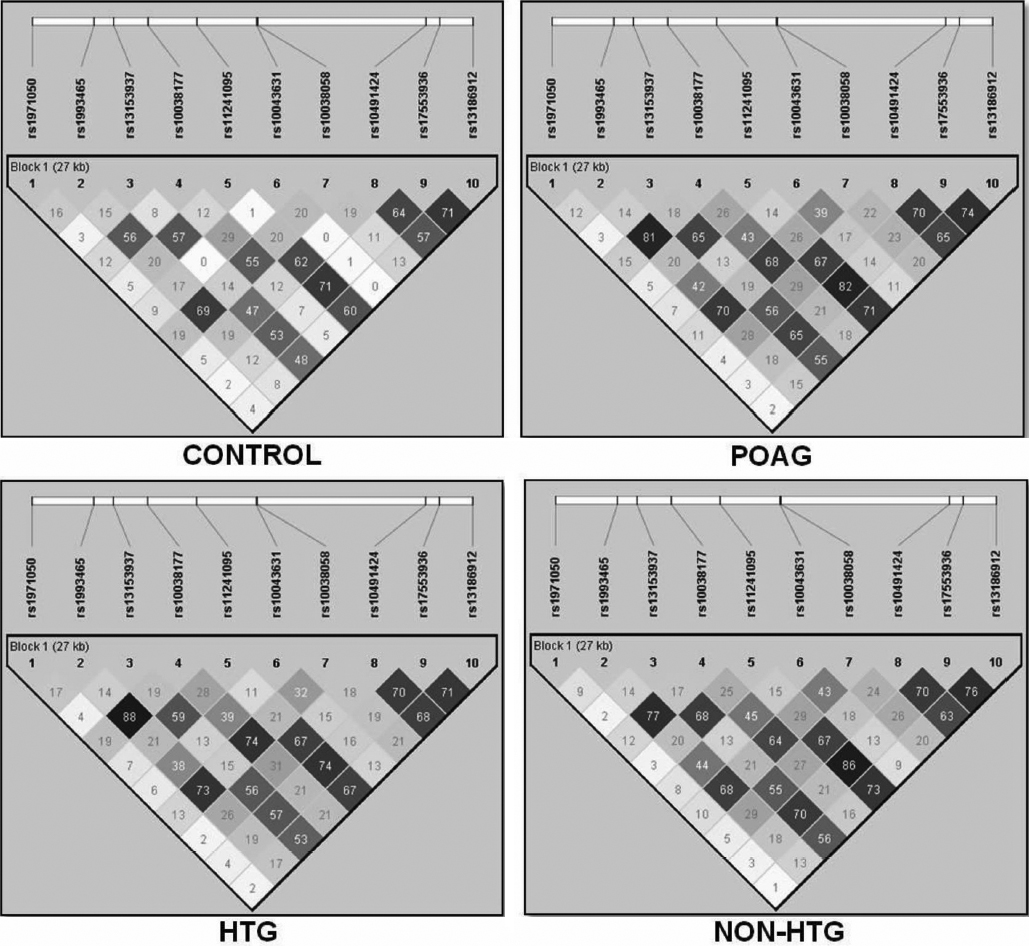
LD pattern (r^2^) of the ten SNPs in *WDR36* in the patient and the control groups. The extent of LD is lowered as the shading lightens (calculated with Haploview 4.1 using standard color schemes).

With the exception of rs1993465 and rs10038177 in the HTG and the POAG patient groups, and rs11241095 and rs17553936 in the POAG and the non-HTG patient groups (Figure 2), no significant LD was observed between the SNPs. All the SNPs were found to be in HW equilibrium.

### *WDR36* haplotype variation among POAG patients and controls

After the haplotype analysis was conducted, a total of 24 different types of haplotype were obtained. Among the different haplotypes TGGTATGTAA, TGATGTGCGT, TAGCACATAA, and CAGCACATAA were found to have a frequency of >5% in both the patient and the control groups. The haplotype TAGCACATAA was found to be associated with the POAG, the HTG, and the non-HTG groups, but these associations were not sustained after Bonferroni correction for multiple testing (Table 6).

**Table 6:** Haplotype frequency of *WDR36* in patient and control groups.

**Haplotype**	**Patient category**	**Frequency in patients (no. of chromosomes)**	**Frequency in controls (no. of chromosomes)**	**p-value**	**OR (95%CI)**	**Bonferroni corrected p-value**
TGGTATGTAA	POAG	0.194 (125)	0.247 (59)	0.077	-	-
	HTG	0.192 (045)		0.159	-	-
	NON-HTG	0.195 (081)		0.118	-	-
TGATGTGCGT	POAG	0.215 (139)	0.198 (47)	0.567	-	-
	HTG	0.181 (042)		0.649	-	-
	NON-HTG	0.233 (096)		0.307	-	-
TAGCACATAA	POAG	0.148 (096)	0.079 (19)	0.007	2.012 (1.206–3.355)	0.168
	HTG	0.170 (039)		0.004	2.329 (1.309–4.141)	0.096
	NON-HTG	0.133 (055)		0.04	1.766 (1.026–3.037)	0.960
CAGCACATAA	POAG	0.056 (036)	0.051 (12)	0.757	-	-
	HTG	0.061 (014)		0.638	-	-
	NON-HTG	0.055 (023)		0.779	-	-

Estimated haplotype frequencies were calculated based on Haploview software. A total of 10 SNPs from phase 1 of the study were used to obtain the haplotype frequencies. The order of SNPs in the haplotype(s) is same as shown in Figure 1. Among 24 different haplotypes obtained from patients and controls, the four haplotypes listed above were found to have a frequency >5% in both patient and control groups. However, no haplotype was found to have any sustainable association with POAG.

## Discussion

Despite studies that have examined *WDR36*’s involvement in POAG in different population groups, no study has yet reported on the role played by this gene in East Indian patients with POAG. Therefore, this study analyzed 10 SNPs in *WDR36* and observed that only a single SNP (c.710+30C>T; rs10038177) was strongly associated with POAG patients who had high IOP.

With the exception of the HTG patients during the first phase of the study, a significant difference in the distribution of age between the POAG cases and the controls was observed. However, no gender bias was observed (Table 1). It is difficult to determine the exact age of onset of POAG because, in many cases, patients seek medical help at an age that is later than the age of onset of the disease, due to its slow progression without symptoms. Therefore, in our study we surrogated the age of onset with the age of diagnosis, which is likely to result in a representation of a relatively older age group compared to the age of most patients when onset of the disease first occurs. Thus, it is likely that the age of patients based on their age at diagnosis is an overestimation when compared to their age at the time of actual onset, which amplifies the difference of the mean age between the cases and the controls. Additionally, overall the mean age of the controls is 50 years and the number of controls is three times higher compared to the HTG cases. Therefore, we argue that the significant difference in age between the HTG cases and the control would not confound our finding.

The role of *WDR36* in POAG still remains elusive; however, from published literature it is known that this gene probably plays an important role in cell survival by virtue of its rRNA processing functions [22,27,28]. Wdr36 has been hypothesized to maintain the homeostasis of retinal ganglion cells (RGC) and the mutant protein was found to reduce the growth of RGC isolated from mouse cells in culture. Furthermore, it led to retinal degeneration in a transgenic mouse expressing mutant protein. Deletion of *WDR36* leads to pre-implantation embryonic lethality in mice specimens and causes apoptotic cell death in a cultured trabecular meshwork cell line [29,30].

The implicated SNP (rs10038177) is located in the proximity of exon 5. Although in silico analysis did not predict any aberrant biologic event due to this intronic variant, it seems reasonable that this SNP, in a less likely event, could directly influence the abnormal gene expression or might be in LD with other yet unidentified variants, which might be responsible for influencing the gene function. However, direct sequencing of *WDR36* exon 5 did not yield any new suspect variant that might be located in the genomic region beyond the coding sequence investigated.

There are conflicting reports regarding the association of rs10038177 (c.710+30C>T) with glaucoma [16,18,31]. An analysis of published data revealed that this SNP was found to be associated with HTG and JOAG cases in a USA study [18]. However, other studies focusing on Caucasian populations did not reveal any association between this SNP and POAG [22,23]. Interestingly, contrary to our observation, the T-allele has been reported to be associated with HTG-cases in one study that examined the Chinese population [16], while in another study no association was observed [31].·Such a difference in observation might be due to the variable influence of multiple other factors associated with POAG in the population groups that these two studies considered. However, lack of information about the ethnicity and interrelationship between these populations limits further analysis of the data.

In conclusion, our study reveals a strong association of a single SNP (rs10038177) in *WDR36* with an HTG patient cohort. No haplotype was found to have any significant association with the POAG cases. However, these observations will remain tentative until similar experiments are conducted on additional and larger cohorts in the same population groups; only then can the initial observations including this study be truly validated.
